# Clinical characteristics, outcomes and regional variations of acquired valvular heart disease patients undergoing cardiac surgery in China

**DOI:** 10.1186/s12872-022-02612-x

**Published:** 2022-04-21

**Authors:** Hongyuan Lin, Jianfeng Hou, Jiamiao Gong, Yongjian Wu, Zhe Zheng

**Affiliations:** Cardiac Surgery Centre, Fuwai Hospital, Chinese Academy of Medical Sciences and Peking Union Medical College, No. 167, North Lishi Street, Xicheng District, , Beijing, 100037 China

**Keywords:** Valvular heart disease, VHD, Surgery, Regional variation, RSMR

## Abstract

**Aims:**

To characterize surgical valvular heart diseases (VHDs) in China and disclose regional variations in VHD surgeries by analyzing the data derived from the Chinese Cardiac Surgery Registry (CCSR).

**Methods and results:**

From January 2016 to December 2018, we consecutively collected the demographic information, clinical characteristics and outcomes of 38,131 adult patients undergoing valvular surgery in China. We sought to assess the quality of VHD surgery by examining in-hospital deaths of all patients from 7 geographic regions. Using a hierarchical generalized linear model, we calculated the risk-standardized mortality rate (RSMR) of each region. By comparing VHD characteristics and RSMRs, we pursued an investigation into regional variations. The mean age was 54.4 ± 12.4 years, and 47.2% of the patients were females. Among cases, the number of aortic valve surgeries was 9361 (24.5%), which was less than that of mitral valve surgeries (n = 14,506, 38.0%). The number of concurrent aortic and mitral valve surgeries was 6984 (18.3%). A total of 4529 surgical VHD patients (11.9%) also underwent coronary artery bypass grafting (CABG) surgery. The overall in-hospital mortality rate was 2.17%. The lowest RSMR, 0.91%, was found in the southwest region, and the highest RSMR, 3.99%, was found in the northeast.

**Conclusion:**

Although the overall valvular surgical mortality rate in large Chinese cardiac centers was in line with high-income countries, there were marked regional variations in the characteristics and outcomes of surgical VHD patients across China.

**Supplementary Information:**

The online version contains supplementary material available at 10.1186/s12872-022-02612-x.

## Background

The prevalence of valvular heart disease (VHD) is growing worldwide as a consequence of prolonged survival and the aging population [1]. Attributed to the improved health care coverage and upgraded medical technology [2], the mortality rate of VHD has declined over the years [2, 3]. However, as one of the most common cardiac surgical diseases [4], VHD is creating the next epidemic [5] and has great persisting burden and challenges [4, 6]. A population-based study performed in the US (n = 11,911), showed the prevalence of moderate or severe VHD was estimated at 2.5% and increased markedly after the age of 65 [7]. Another cohort study [8] held in the UK showed that newly identified VHD was found in 1269 of the first 2500 enrolled participants aged > 65 years (50.8%), 11.3% of the cohort was clinically significant and was more common in lower socioeconomic classes which indicating a regional variation of VHD prevalences. Attributed to the highly technical demands of cardiac surgery, prominent regional variations in surgical outcomes were also documented [9]. For example, in the US, the risk-standardized mortality rates (RSMR) of coronary artery bypass grafting (CABG) surgery was 2.25% in southern region, much higher than the Northeastern (1.63%) [9]. Similarly, in China, eastern region had the lowest CABG RSMR of 1.6%, whereas central region had the highest RSMR of 2.5% [10]. To achieve consistent high-quality surgical care throughout the whole country, nationwide studies are needed to track the quality of cardiac surgeries across regions. However, unlike CABG, there is a lack of studies on the regional variations in VHD surgical outcomes. Therefore, we sought to assess the quality of adult VHD surgery by examining the in-hospital mortality rates based on the Chinese Cardiac Surgery Registry (CCSR) [11], which is the largest cardiac surgery database in China.

## Methods

### Data source

Our study was based on the CCSR, a multicenter registry. The registry is overseen by a steering committee that includes cardiac surgeons and researchers from Fuwai Hospital and the National Center of Cardiovascular Diseases (NCCD). The data related to VHD surgery in this database came from a total of 94 hospitals, which were grouped into 7 regions (northeast, north, east, central, south, southwest and northwest) according to their geographical locations. Each participating hospital had a cardiac surgery volume of > 100 operations/year and was required to record cases using a standardized case report form (CRF). These sites are the leading cardiac centers in local regions and have many features that are common among large cardiac care centers in China. According to the annual surveys conducted by the Chinese Society of Extracorporeal Circulation, we estimate that our database has included approximately 30–40% of all valve operations and represents performance in large cardiac centers [11]. Every 6 months, two researchers randomly checked 5–10% of the reported cases for auditing purposes. For cases with incomplete information or any data problems, the relevant participating units were required to rectify the issues to ensure the authenticity and integrity of the data. In addition to auditing the completeness of the data, the quality appraisal has been only performed in CABG [10, 12], lacking in VHD surgery.

### Patients and characteristics

**Fig. 1:**
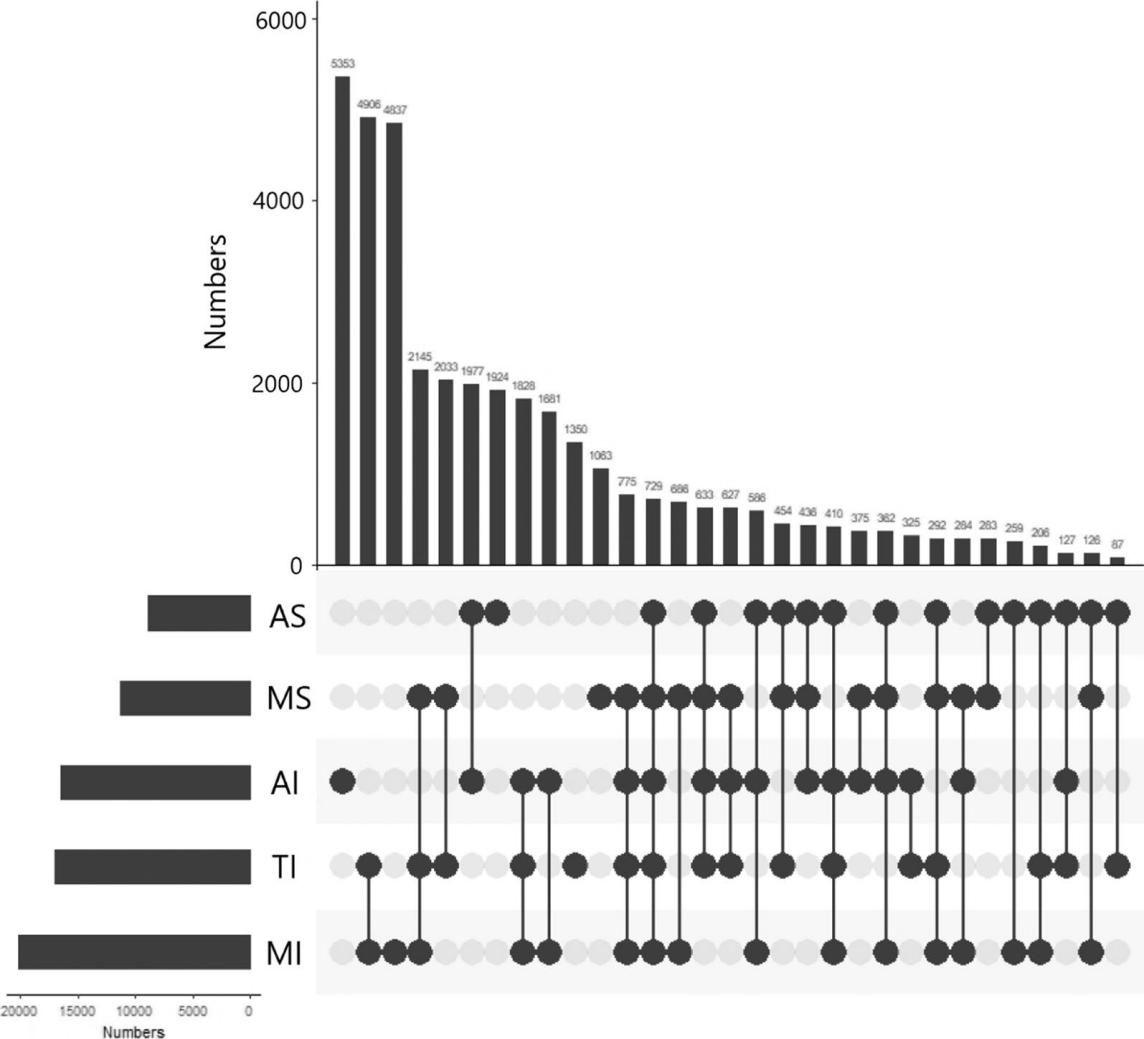
The summary of VHD lesion types. AS: aortic stenosis; AI: aortic insufficiency; MS: mitral stenosis; MI: mitral insufficiency; TI: tricuspid insufficiency

**Fig. 2:**
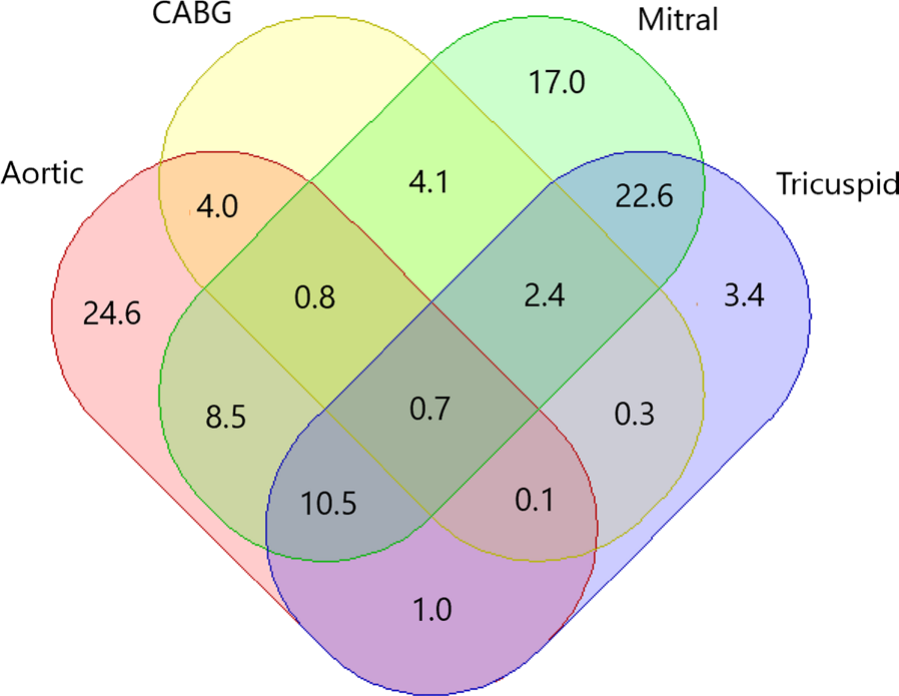
The percentages of different surgical methods and their combinations. Aortic: aortic valvular replace or repair; Mitral: mitral valvular replace or repair; CABG: coronary artery bypass grafting; Tricuspid: tricuspid valvular replace or repair

**Fig. 3:**
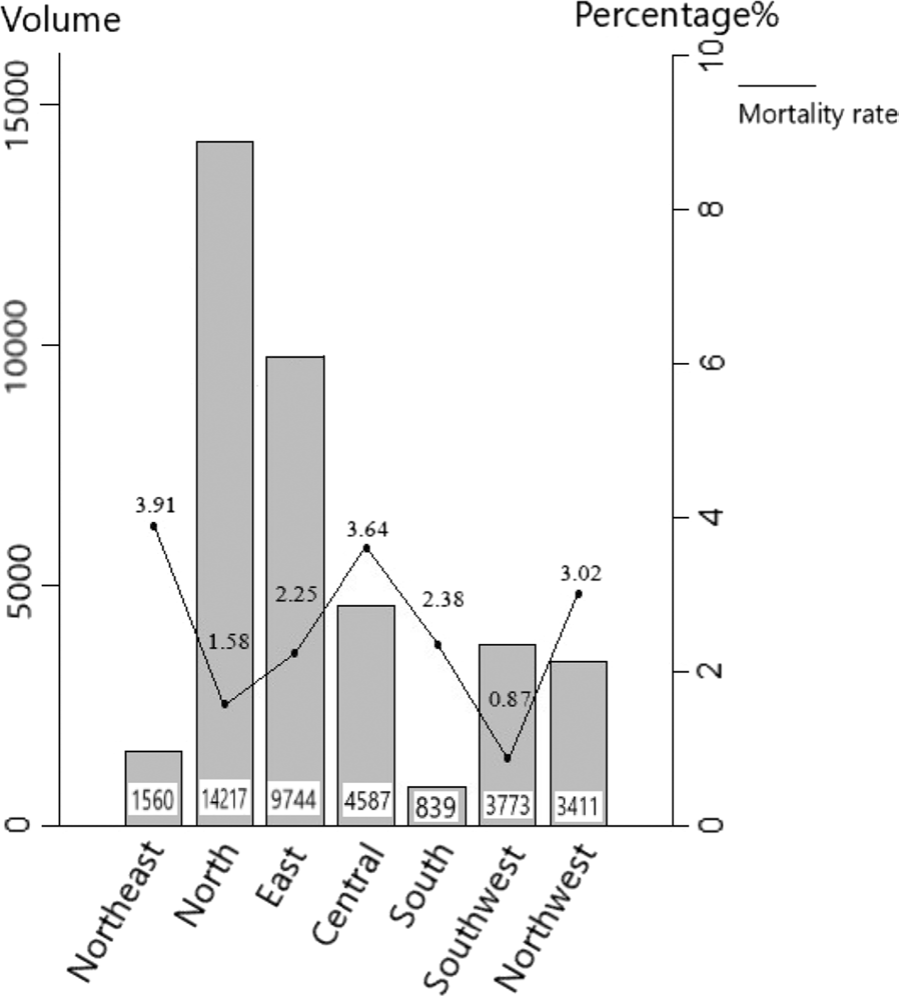
Each region’s sample size and observed in-hospital mortality

We identified 43,877 patients who underwent valvular surgery in the CCSR database between January 1, 2016, and December 31, 2018. Among these patients, we excluded a total of 5746 patients who were under age 18 and enrolled the final study population with a sample size of 38,131. We collected risk factors, which were listed in Table 1, including demographic data, characteristics of valvular lesions, preoperative risk factors, operative information, and important postoperative parameters. We also summarized all types of surgical valvular lesions, as shown in Fig. 1. The surgical methods were presented in Fig. 2. The sample sizes of the 7 regions and the corresponding observed in-hospital mortality rates were shown in Fig. 3.

**Table 1:** Demographics and risk factors of each region (median [IQR] or frequency (percentages))

Variables	Northeast (n = 1560)	North (n = 14,217)	East (n = 9744)	Central (n = 4587)	South (n = 839)	Southwest (n = 3773)	Northwest (n = 3411)	Overall (n = 38,131)
*Patient related*								
Age*	56.94 [49.75, 63.50]	55.68 [46.95, 63.70]	58.19 [49.41, 65.54]	54.27 [46.59, 62.39]	54.84 [46.10, 63.44]	52.02 [45.51, 60.33]	53.26 [46.28, 60.55]	55.31[47.29, 63.50]
Female	732 (46.9)	6559 (46.1)	4689 (48.1)	2118 (46.2)	368 (43.9)	2113 (56.0)	1436 (42.1)	18,015 (47.2)
BMI	23.51 [21.33, 25.80]	23.44 [21.11, 25.78]	22.89 [20.76, 25.00]	22.60 [20.70, 24.84]	22.37 [20.08, 24.57]	22.51 [20.50, 24.69]	22.86 [20.71, 25.00]	23.03 [20.82, 25.31]
BSA(m^2^)*	1.67 [1.55, 1.81]	1.68 [1.55, 1.81]	1.62 [1.50, 1.75]	1.64 [1.53, 1.76]	1.59 [1.48, 1.71]	1.56 [1.46, 1.68]	1.66 [1.55, 1.78]	1.64 [1.53, 1.78]
Diabetes mellitus	131 (8.4)	1046 (7.4)	732 (7.5)	271 (5.9)	73 (8.7)	162 (4.3)	152 (4.5)	2567 (6.7)
Hypertension	447 (28.7)	4388 (30.9)	2951 (30.3)	1090 (23.8)	180 (21.5)	439 (11.6)	655 (19.2)	10,150 (26.6)
eGFR*	91.98 [76.84, 102.39]	88.59 [73.38, 100.35]	90.66 [75.20, 101.65]	95.09 [79.86, 105.88]	87.87 [70.70, 102.00]	94.09 [79.63, 104.95]	86.79 [69.40, 102.36]	90.70[74.69, 102.22]
CKD	20 (1.3)	874 (6.1)	151 (1.5)	51 (1.1)	11 (1.3)	16 (0.4)	10 (0.3)	1133 (3.0)
Dialysis*	1 (0.1)	69 (0.5)	29 (0.3)	12 (0.3)	1 (0.1)	4 (0.1)	1 (0.0)	117 (0.3)
COPD*	14 (0.9)	106 (0.7)	72 (0.7)	83 (1.8)	9 (1.1)	31 (0.8)	49 (1.4)	364 (1.0)
Previous stroke*	31 (2.0)	557 (3.9)	496 (5.1)	292 (6.4)	51 (6.1)	93 (2.5)	106 (3.1)	1626 (4.3)
Extracardiac arteriopathy	21 (1.3)	270 (1.9)	390 (4.0)	145 (3.2)	18 (2.1)	6 (0.2)	47 (1.4)	897 (2.4)
*Heart related*								
NYHA III or IV*	1261 (80.8)	5804 (40.8)	5042 (51.7)	2331 (50.8)	514 (61.3)	2846 (75.4)	2173 (63.7)	19,971 (52.4)
Arrhythmia	523 (33.5)	1719 (12.1)	2989 (30.7)	1287 (28.1)	298 (35.5)	1267 (33.6)	858 (25.2)	8941 (23.4)
Critical status*	16 (1.0)	53 (0.4)	80 (0.8)	21 (0.5)	14 (1.7)	29 (0.8)	22 (0.6)	235 (0.6)
Previous myocardial infarction	41 (2.6)	427 (3.0)	127 (1.3)	90 (2.0)	32 (3.8)	31 (0.8)	41 (1.2)	789 (2.1)
Previous cardiac surgery*	42 (2.7)	1031 (7.3)	422 (4.3)	229 (5.0)	58 (6.9)	90 (2.4)	109 (3.2)	1981 (5.2)
LVEF*								
LVEF ≥ 50%	1407 (90.2)	13,429 (94.5)	8633 (88.6)	4094 (89.3)	742 (88.4)	3049 (80.8)	2869 (84.1)	34,223 (89.8)
35% ≤ LVEF < 50%	136 (8.7)	692 (4.9)	992 (10.2)	457 (10.0)	89 (10.6)	641 (17.0)	518 (15.2)	3525 (9.2)
LVEF < 35%	17 (1.1)	96 (0.7)	119 (1.2)	36 (0.8)	8 (1.0)	83 (2.2)	24 (0.7)	383 (1.0)
Left main stenosis*	27(1.7)	194(1.4)	299(3.1)	74(1.6)	36(4.3)	54(1.4)	43(1.3)	727(1.9)
AS	427 (27.4)	2606 (18.3)	2352 (24.1)	1106 (24.1)	221 (26.3)	1260 (33.4)	885 (25.9)	8857 (23.2)
Severe AI	230 (14.7)	742 (5.2)	1663 (17.1)	862 (18.8)	165 (19.7)	757 (20.1)	749 (22.0)	5168 (13.6)
MS	484 (31.0)	2764 (19.4)	3062 (31.4)	1480 (32.3)	331 (39.5)	2049 (54.3)	1099 (32.2)	11,269 (29.6)
Severe MI	572 (36.7)	1015 (7.1)	2477 (25.4)	1392 (30.3)	244 (29.1)	877 (23.2)	544 (15.9)	7121 (18.7)
Severe TI*	185 (11.9)	368 (2.6)	1003 (10.3)	512 (11.2)	107 (12.8)	447 (11.8)	205 (6.0)	2827 (7.4)
RHD	547 (35.1)	4504 (31.7)	4790 (49.2)	2218 (48.4)	345 (41.1)	2137 (56.6)	1526 (44.7)	16,067 (42.1)
Endocarditis	83 (5.3)	439 (3.1)	317 (3.3)	194 (4.2)	54 (6.4)	65 (1.7)	99 (2.9)	1251 (3.3)
*Operation related*								
Non-elective surgery*	17 (1.1)	225 (1.6)	91 (0.9)	73 (1.6)	17 (2.0)	19 (0.5)	70 ( 2.1)	512 ( 1.3)
Aortic aneurysm operation*	10(0.6)	317(2.2)	359(3.7)	198(4.3)	50(5.9)	115(3.0)	343(10.0)	1392(3.7)
CABG*	155 (9.9)	2219 (15.6)	1097 (11.3)	674 (14.7)	141 (16.8)	181 (4.8)	209 ( 6.1)	4676 (12.3)

IQR: interquartile range; BMI: body mass index; BSA: body surface area; eGFR: estimated glomerular filtration rate; CKD: chronic kidney disease; COPD: chronic obstructive pulmonary disease; NYHA: New York heart association; LVEF: left ventricular ejection fraction; AS: aortic valvular stenosis; AI: aortic valvular insufficiency; MS: mitral valvular stenosis; MI: mitral valvular insufficiency; TI: tricuspid insufficiency; RHD: rheumatic heart disease; CABG: coronary artery bypass grafting

*Independent risk factors which were included in the HGLM for risk adjustment

### Statistical analysis

R software version 4.0.2 was used for statistical analyses. GraphPad Prism for Windows version 6.0 was used to create graphs. Categorical variables are reported as frequencies (percentages) and were compared between groups by chi-square test. The Kolmogorov–Smirnov test was adopted for normality testing. Continuous variables were reported as the means ± SD or as the median with interquartile range (as median [IQR]) and were compared with one-way ANOVA or the Kruskal–Wallis H test as appropriate. A P value of less than 0.05 was considered statistically significant.

For the CCSR database, date of birth and in-hospital survival status (death or not) were 100% complete, and the majority of key variables were > 98% complete, with the exception of length of stay (LOS, 85% complete). Values such as “Unknown” were set to null.

Using the hierarchical generalized linear model (HGLM), we estimated a random intercept risk model relating the log-odds of in-hospital mortality to patient risk factors for the study sample [13]. The candidate variables (Table 1) used for risk adjustment were selected on the basis of clinical knowledge and previous studies [8–10, 12]. The descriptions of variables were listed in Table 2. After univariate screening and multivariate hierarchical logistic regression (Additional file 1: Tables S1, S2), 15 variables were included in the final HGLM for risk adjustment (Table 1). We next calculated the risk-standardized mortality rates (RSMRs), defined as the ratio of the number of “predicted” to “expected” deaths, multiplied by the national unadjusted raw mortality rate [14]. The expected number of deaths for each region was estimated by applying the estimated regression coefficients of the HGLM to the patient characteristics of each patient in each region, adding the average of the region-specific intercepts, and after transformation, summing the data of all patients in the region to obtain the count. The predicted number of deaths was calculated by applying the estimated regression coefficients to the patient characteristics of the patients in each region, adding the region-specific intercept (representing the baseline mortality risk in the specific region), and after transformation, summing all the data for patients in the region to obtain a predicted count. We then used the bootstrapping sampling method (repeated 1000 times with random sampling of 10% of the sample for each region) to calculate a 95% confidence interval for each region’s RSMR.

**Table 2:** Description of variables

Variables	Description
Age	–
Female	–
BMI	Body mass index
BSA(m^2^)	Body surface area
Diabetes mellitus	Documented past history or fulfilled the criteria of WHO 1999
Hypertension	Documented past history or SBP > 140 mmHg and/or DBP > 90 mmHg
eGFR	Estimated glomerular filtration rate
CKD	Documented past history or fulfilled the criteria of KDIGO 2012
Dialysis	Documented past history
COPD	Long-term use of bronchodilators or steroids for lung disease
Previous stroke	Documented past history of coma ≥ 24 h or central nervous system dysfunction ≥ 72 h
Extracardiac arteriopathy	Any one or more of the following: claudication, carotid occlusion or > 50% stenosis, previous or planned intervention on the abdominal aorta, and limb arteries or carotids
NYHA III or IV	NYHA classification
Arrhythmia	Atrial fibrillation, flutter or atrioventricular block within 2 weeks before operation
Critical status	Any one or more of the following occurring preoperatively: ventricular tachycardia or fibrillation or aborted sudden death; cardiac massage; ventilation before arrival in the anaesthetic room; inotropes; intra-aortic balloon counterpulsation or ventricular-assist device before arrival in the anaesthetic room; acute renal failure (anuria or oliguria < 10 ml/h)
Previous myocardial infarction	Documented past history
Previous cardiac surgery	One or more previous major cardiac operation involving opening the pericardium
LVEF	Assessed by echocardiography (measured before surgery)
Left main stenosis	Left main coronary artery stenosis > 50%
AS	Assessed by echocardiography (measured before surgery)
Severe AI	Assessed by echocardiography (measured before surgery)
MS	Assessed by echocardiography (measured before surgery)
Severe MI	Assessed by echocardiography (measured before surgery)
Severe TI	Assessed by echocardiography (measured before surgery)
RHD	Documented past history
Endocarditis	Documented past history
Non-elective surgery	Not routine admission for operation
Aortic aneurysm operation	Combined with aortic aneurysm (or dissecting aneurysm) operation
CABG	Combined with CABG operation

BMI: body mass index; BSA: body surface area; WHO: world health organization; SBP: systolic blood pressure; DBP: diastolic blood pressure; eGFR: estimated glomerular filtration rate; CKD: chronic kidney disease; KDIGO: Kidney Disease Improving Global Outcomes; COPD: chronic obstructive pulmonary disease; NYHA: New York heart association; LVEF: left ventricular ejection fraction; AS: aortic valvular stenosis; AI: aortic valvular insufficiency; MS: mitral valvular stenosis; MI: mitral valvular insufficiency; TI: tricuspid insufficiency; RHD: rheumatic heart disease; CABG: coronary artery bypass grafting

## Results

### Variation of demographics and risk factors

Table 1 demonstrated demographics and risk factors of our cohort. We could find that the overall prevalence of rheumatic heart disease (RHD) was 42.1% and a prominent regional variation was revealed. The southwest region had the highest prevalence of 56.6%, whereas the northern region had the lowest prevalence of 31.7%. Moreover, prevalences of some other independent risk factors of VHD surgical mortality also varied regionally. For example, NYHA class III or IV was more common in the northeast (80.8%), and the lowest in the north region (40.8%). The highest prevalence of CKD was in the north (6.1%) and the lowest was in the northwest (0.3%). The prevalence of concurrent CABG was highest in the south (16.8%) and lowest in the southwest (4.8%).

### Characteristics of VHD lesions

All the types of surgical VHD lesions are shown in Fig. 1. From these data, we identified that the top three prevalent lesion types were isolated aortic regurgitation (n = 5353), mitral regurgitation combined with tricuspid regurgitation (n = 4906), and isolated mitral regurgitation (n = 4837). In general, valvular insufficiency (n = 33,889) was more prevalent than stenosis (n = 16,859) and there were more mitral lesions (n = 26,016) than aortic lesions (n = 20,392).

### Surgical methods selection

The application of surgical methods was summarized in Fig. 2, in which we found the following: 1) The number of mitral valve surgeries was 25,395 (66.6%), greater than that of aortic valve surgery (n = 19,142, 50.2%). 2) 4,690 cases (12.3%)of the VHD surgeries were performed concomitant with CABG. 3) Compared with aortic valve surgery (24.5%), mitral valve surgery had a greater propensity (54.4%) to be combined with a tricuspid valve procedure.

### Comparison of outcomes between regions

**Fig. 4:**
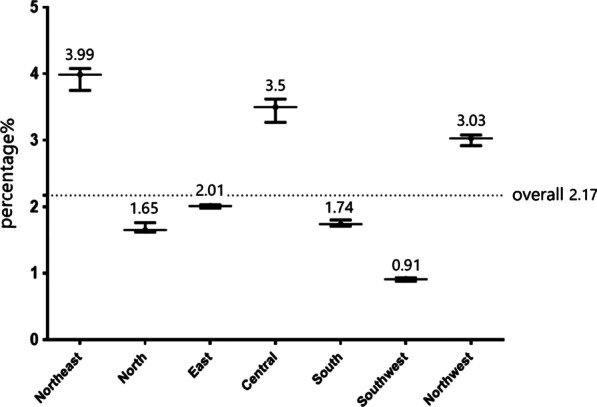
Each region’s risk standardized in-hospital mortality rate with 95% confidence interval

The observed overall mortality rate of the whole sample was 2.17%. The sample size of each region and observed in-hospital mortality were compared in Fig. 3. The data clearly showed that more cases were enrolled in the northern (n = 14,217, 37.3%) and eastern regions (n = 9744, 25.6%). With respect to the observed mortality rates, North (1.58%) and Southwest (0.87%) performed better than the other regions. Figure 4 showed the corresponding RSMRs of different regions, which could make the identification of regional variations in RSMRs easy to discern. Roughly, the RSMRs in the north (1.65%), east (2.01%) and southwest (0.91%) regions were significantly lower than those in the northeast (3.99%), central (3.5%) and northwest (3.03%) regions.

## Discussion

In this contemporary report of valvular surgery in China, we assessed clinical characteristics and in-hospital mortality rates and demonstrated differences across regions, with some regions performing significantly better and worse than the overall average. Our findings provide evidence-based information about the performance of VHD surgery across regions. To the best of our knowledge, this is the first study to report regional variations of VHD surgery in China. The following advantages of performing this nationwide study on the quality of surgery for VHDs across regions are realized: (1) The surgical mortality rates and other characteristics of all regions were compared; therefore, surgeons in corresponding regional centers can identify relevant problems. (2) As mentioned above, given that CCSR data is representative and generalizable in the field of cardiac surgery in China, the study established a benchmark for other cardiac centers to reference. (3) Disclosing the regional variations and providing important references will help optimize the allocation of medical resources.

In view of the characteristics of VHD lesions, in surgical cases, regurgitation disease was more common than stenosis (Fig. 1), whereas insufficient lesions were more often associated with degenerative diseases, which might be more prevalent in aging populations and high-income countries [15]. In our study, which was conducted in a middle-income country, rheumatic heart diseases (RHDs) accounted for 42.1% of the surgical cases, whereas RHDs accounts for only 4% of the VHD cases in western or northern Europe, and 12–14% in eastern or southern Europe [15]. However, worldwide, RHD remains the most prevalent form of VHD and contributes to substantial premature mortality [16] and reduced quality of life [1]. In other low and middle income countries, RHD remains the leading cause of valvular surgeries [7, 17]. Ribeiro et al. [18] reported that 60.3% of valvular surgeries in a Brazilian urban center were RHD cases. In north Africa, the proportion of RHD was as high as 72% [15]. Apparently, the prevalence of RHD is closely related to the socioeconomic level. We found that the southwest region had the largest proportion of RHD, accounting for 56.6% of the surgical cases, probably due to its relatively lower socioeconomic level.

In current study, the overall in-hospital mortality rate of VHD surgery was 2.17%, which was indeed slightly better than that in some high-income countries, such as the US (2.64%). M. Bowdish et al. [4] released an update of the Society for Thoracic Surgeons (STS) adult cardiac surgery database in 2021, showing that the in-hospital mortality rate of VHD surgery was 2.64% in 2019 (n = 66,123, including the 6 most commonly performed operations). Therefore, with respect to in-hospital mortality, VHD surgery levels at large cardiac centers in China have improved remarkably in recent years and are in line with those in some high-income countries. In spite of a low overall mortality rate, there was great variation in RSMRs across regions with the highest RSMR of 3.99% in northeast, four-fold higher than the lowest of 0.91% in southwest.

By establishing the HGLM model, we ultimately selected 15 independent risk factors associated with in-hospital mortality for risk adjustment (Table 1). Identification of these risk factors before surgery is of great significance to improve surgical outcomes in clinical practice. The levels or exposure rates of these risk factors also varied across geographic regions, indicating regional variation in the inherent severity of VHDs. For instance, in the north region, there were more chronic kidney disease (CKD) patients needing dialysis (0.5%, with an overall rate of 0.3%) and more concomitant CABG surgeries (15.6%, with an overall rate of 12.3%). Similarly, in the northeast region, the proportion of patients with New York Heart Association (NYHA) class III or IV was 80.8%, which was much higher than that in the other regions. The regional variation in risk factors might arise from disparities in lifestyle and metabolic features [19, 20]. As China is a country with the largest population in the world and 56 ethnic groups, its geographic regional variations are more evident, as are VHD features and levels of medical care. A nationwide study [2] from China showed that the age-standardized prevalence of cardiovascular disease (CVD) including VHD increased by 14.7% from 1990 to 2016. The relative burden of CVD varied widely at the provincial level. A nearly sixfold difference in the total burden of CVD persists among provinces. This suggested a deteriorating balance in cardiovascular health within China.

In addition to regional variations in VHD characteristics, indications and contraindications for surgery might be controlled differently across regions, which was exemplified by data obtained from the southwest region. In the southwest, the observed mortality rate and RSMR were significantly lower than those of other regions. However, by comparing the preoperative characteristics of the surgical cases, we ascertained that the rates of preoperative dialysis, stroke, previous cardiac surgery, nonelective surgery and concomitant CABG in the southwest were also lower than those in other regions (Table 1). It was suggested that the surgical indications in this region were more strictly controlled. In other words, patients in the southwest who underwent surgery were less sick than their counterparts in other regions. Therefore, the lower RSMR of this region might not indicate a truly higher level of medical care.

The socioeconomic development of China is not balanced across regions: development in the north and east regions is the highest, whereas development in the northwest region is the lowest. Consequently, more high-quality medical resources are concentrated in the north and east regions, resulting in their larger volumes of surgeries documented in this study (Fig. 3) and lower RSMRs (Fig. 4). This result suggested that richer regions could have better surgical outcomes. Dominique Vervoort et al. [21] conducted a survey suggesting that disparities exist between and within world regions, with a positive correlation between a nation’s economic status and access to cardiac surgery. Moreover, the availability of adult and pediatric cardiac surgical workforces is scarce in low- and middle-income countries [21]. Similar results were reported by Mehaffey and colleagues in a study [22] on CABG, suggesting that patients in distressed communities were at increased risk for adverse events and death after CABG.

The quality of surgery for VHD, one of the most common cardiac diseases, could, to some degree, represent the level of cardiac surgery in a region. There existed predominant variations in the use of surgical interventions by country income level [16, 17]. China, with the largest population in the world, has the most cases of VHD, which could cause a high societal burden [5]. Analyzing and summarizing the national surgical data of VHD could provide an important reference for policy-making aimed at improving national medical care.

### Limitations

Despite several strengths of the CCSR data, including a comparatively large sample.

size, generalizability to the Chinese population, and detailed information on surgeries, our study was limited by the lack of follow-up data on survival and other major outcomes. Including follow-up data may have provided more comprehensive insights into the quality of medical care.

## Conclusion

Although the overall valvular surgical mortality rate in large Chinese cardiac centers was in line with high-income countries, there were marked regional variations in the characteristics and outcomes of surgical VHD patients across China.

## Supplementary Information

**Additional file 1.** Summary of multivariate hierarchical logistic regression (HGLM model) and RSMRs in different regions.

## Data Availability

The CCSR registry is not publicly available, but the datasets used and/or analysed during the current study are available from the corresponding author on reasonable request.
