# Supplementary material for: Clinical characteristics, outcomes and regional variations of acquired valvular heart disease patients undergoing cardiac surgery in China
Source: BMC Cardiovasc Disord. 2022 Apr 21;22:188. doi: 10.1186/s12872-022-02612-x (PMC9026662; doi:10.1186/s12872-022-02612-x)
Supplement: Supplementary file 1 — Additional file 1. Summary of multivariate hierarchical logistic regression (HGLM model) and RSMRs in different regions. [file 12872_2022_2612_MOESM1_ESM.docx]

Supplement table 1. Summary of multivariate hierarchical logistic regression (HGLM model)

| Variables | Estimate coefficient | Std. Error | t-value | P value |
| --- | --- | --- | --- | --- |
| Fixed effects estimates | | | | |
| (Intercept) | -2.7365838 | 0.2718033 | -10.068 | <0.001 |
| BSA | -0.9181664 | 0.0933015 | -9.841 | <0.001 |
| eGFR | -0.0100194 | 0.0007687 | -13.034 | <0.001 |
| Age>60 | 0.3511006 | 0.0203660 | 17.240 | <0.001 |
| Dialysis | 1.1485274 | 0.1499044 | 7.662 | <0.001 |
| COPD | 0.5094479 | 0.0996360 | 5.113 | <0.001 |
| Previous stroke | 0.5932351 | 0.0555731 | 10.675 | <0.001 |
| NYHA III or IV | 0.4079803 | 0.0341709 | 11.939 | <0.001 |
| Critical status | 1.1017629 | 0.1105421 | 9.967 | <0.001 |
| Previous cardiac surgery | 0.8638533 | 0.0508130 | 17.001 | <0.001 |
| Left main stenosis | 0.5862107 | 0.0707139 | 8.290 | <0.001 |
| Severe TI | 0.4827506 | 0.0481700 | 10.022 | <0.001 |
| Non-elective surgery | 1.8621607 | 0.0663794 | 28.053 | <0.001 |
| CABG | 0.7382761 | 0.0394062 | 18.735 | <0.001 |
| Aortic aneurysm operation | 0.5895416 | 0.0659048 | 8.945 | <0.001 |
| LVEF<35% | 0.5941222 | 0.0323596 | 18.360 | <0.001 |
| Random effects (region) estimates | | | | |
| Northeast | 0.6454 | 0.2141 |  |  |
| North | -0.2936 | 0.2102 |  |  |
| East | -0.0837 | 0.2100 |  |  |
| Central | 0.5202 | 0.2106 |  |  |
| South | -0.2405 | 0.2245 |  |  |
| Southwest | -0.9046 | 0.2180 |  |  |
| Northwest | 0.3570 | 0.2119 |  |  |

BSA: body surface area; eGFR: estimated glomerular filtration rate; COPD: chronic obstructive pulmonary disease; NYHA: New York heart association; TI: tricuspid insufficiency; CABG: coronary artery bypass grafting; LVEF: left ventricular ejection fraction.

Supplement table 2. Summary of RSMRs in different regions

|  | Northeast  (n=1560) | North  (n=14217) | East  (n=9744) | Central  (n=4587) | South  (n=839) | Southwest  (n=3773) | Northwest  (n=3411) |
| --- | --- | --- | --- | --- | --- | --- | --- |
| Predicted death number | 60.26 | 223.73 | 217.63 | 165.68 | 20.02 | 33.34 | 102.14 |
| Expected death number | 32.79 | 294.56 | 235.43 | 102.65 | 25.00 | 79.75 | 73.12 |
| RSMR | 0.0399 | 0.0165 | 0.0201 | 0.035 | 0.0174 | 0.0091 | 0.0303 |
| Bootstrap 95%CI | 0.0375-0.0408 | 0.0162-0.0176 | 0.0199-0.0202 | 0.0327-0.0362 | 0.0171-0.018 | 0.0088-0.0093 | 0.0292-0.0308 |
| Raw mortality rate=0.0217 | | | | | | | |
